# Cervical Diffuse Idiopathic Skeletal Hyperostosis: Rare Cause of Emergency Tracheostomy

**DOI:** 10.7759/cureus.20925

**Published:** 2022-01-04

**Authors:** Panagiota Kosmidou, Ioannis Karamatzanis, Stavros Angelis, Andreas Anagiotos, Andreas Aspris

**Affiliations:** 1 Otolaryngology - Head and Neck Surgery, Mediterranean Hospital of Cyprus, Limassol, CYP; 2 Otolaryngology - Head and Neck Surgery, University of Patras Medical School, Patras, GRC; 3 Otorinolaryngology, University of Nicosia Medical School, Nicosia, CYP; 4 Surgical Anatomy, National and Kapodistrian University of Athens Medical School, Athens, GRC; 5 Otorhinolaryngology, Nicosia General Hospital, Nicosia, CYP

**Keywords:** vocal cord paresis, osteophytes, tracheostomy, dysphagia, cervical hyperostosis, diffuse idiopathic skeletal hyperostosis

## Abstract

Diffuse idiopathic skeletal hyperostosis (DISH) is a rare and potentially life-threatening syndrome. We present the case of a patient complaining about severe dyspnoea and diagnosed with vocal cord paresis. An emergency tracheotomy was performed to restore his breathing. Diagnostic imaging revealed large mass-occupying cervical osteophytes compressing the larynx. The osteophytes were removed via an anterior cervical approach, and vital signs were normalized. However, postoperatively, a fistula was discovered between the upper part of the oesophagus and the trachea. As a result, a gastrostomy tube had to be placed indefinitely. Literature review confirms the rare frequency of emergency tracheostomy due to DISH syndrome. The aim of the present study is to expand on our knowledge of a rare pathological entity that can frequently be misdiagnosed.

## Introduction

Diffuse idiopathic skeletal hyperostosis (DISH), formerly known as Forestier’s disease, is a rare syndrome defined by the presence of ossification in the axial skeleton and peripheral entheses [1]. The exact epidemiology of the syndrome is unclear, but it predominantly affects males, while incidence increases with age [2]. The hallmark of the disease is the presence of flowing ossification in the anterolateral aspect of the thoracic spine, over at least four contiguous vertebral bodies [3]. The ossifications are more commonly found on the right side of the spine, with the prevailing theory attributing this to the protective barrier formed by the arterial distribution, such as the descending aorta [1].

DISH syndrome poses a variable presentation, ranging from asymptomatic patients to severe back pain and limited spinal motion. The difference in symptoms and their location is linked to the position of the osteophytes, which mainly exert their effect via mass effect [2]. Cervical osteophytes frequently lead to dysphagia, airway obstruction, cervical pain, and difficulty during intubation [4]. Thoracic and lumbar osteophytes are more likely to lead to back pain, limited range of motion (ROM), and increased susceptibility to fractures [5].

We present the case of a patient with cervical osteophytes due to DISH syndrome suffering from airway obstruction and unilateral vocal cord paralysis.

## Case presentation

**Figure 1:**
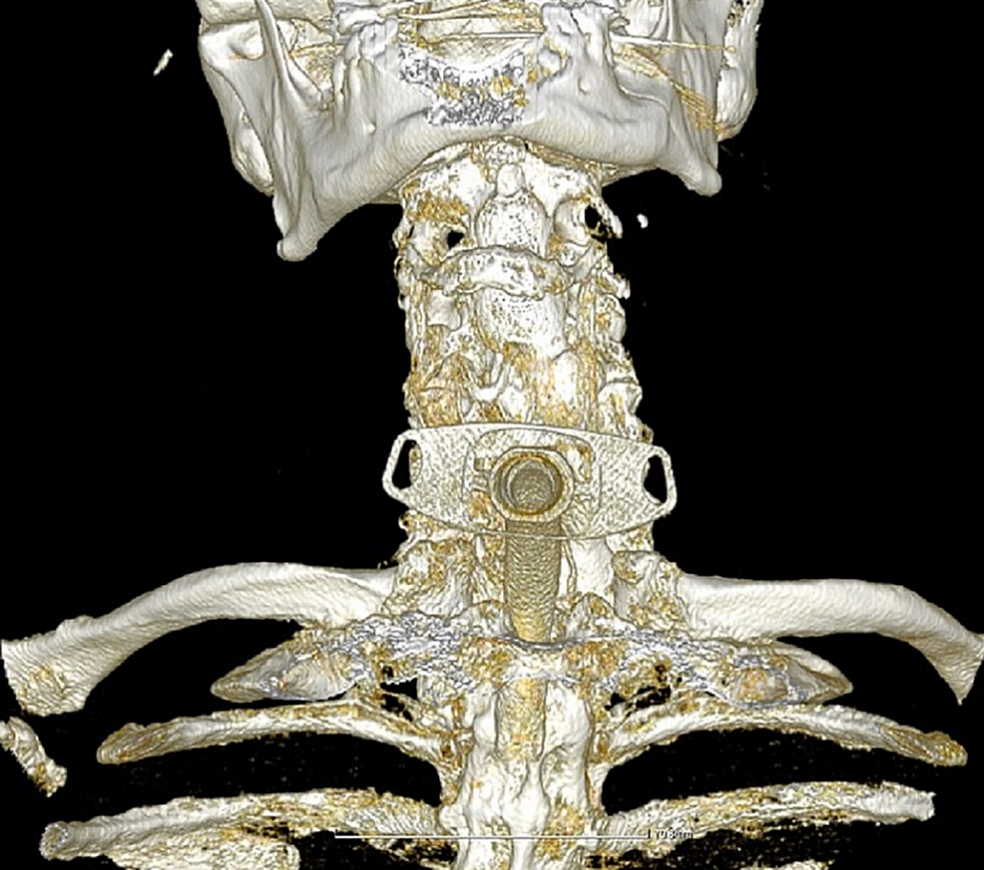
Three-dimensional reconstruction of the tracheostomy.

A 69-year-old male presented to the emergency unit with inspiratory stridor and dyspnoea attributed to obstruction of the upper respiratory system. Endoscopy of the larynx was immediately performed, using a flexible endoscope, where left vocal cord paresis with laryngeal oedema localized to the arytenoids was discovered. Vitals signs recorded upon admission included heart rate of 80 beats/minute, respiratory rate of 40 breaths/minute, and oxygen saturation of 89%. A lifesaving emergency tracheotomy was performed and his oxygen saturation increased to 93% (Figure 1). Τhe patient was admitted to the intensive care unit.

The patient reported no known allergies and no alcohol drinking habits, but a 30-year-pack history of cigarette smoking. Overall, the medical history included arterial hypertension under good control with medication, prostatectomy, and left hip replacement. However, the patient reported difficulty in swallowing liquids and solids for the past year, and intense hoarseness during the last month. He also reported back pain that has been increasing in intensity during the past nine months.

**Figure 2:**
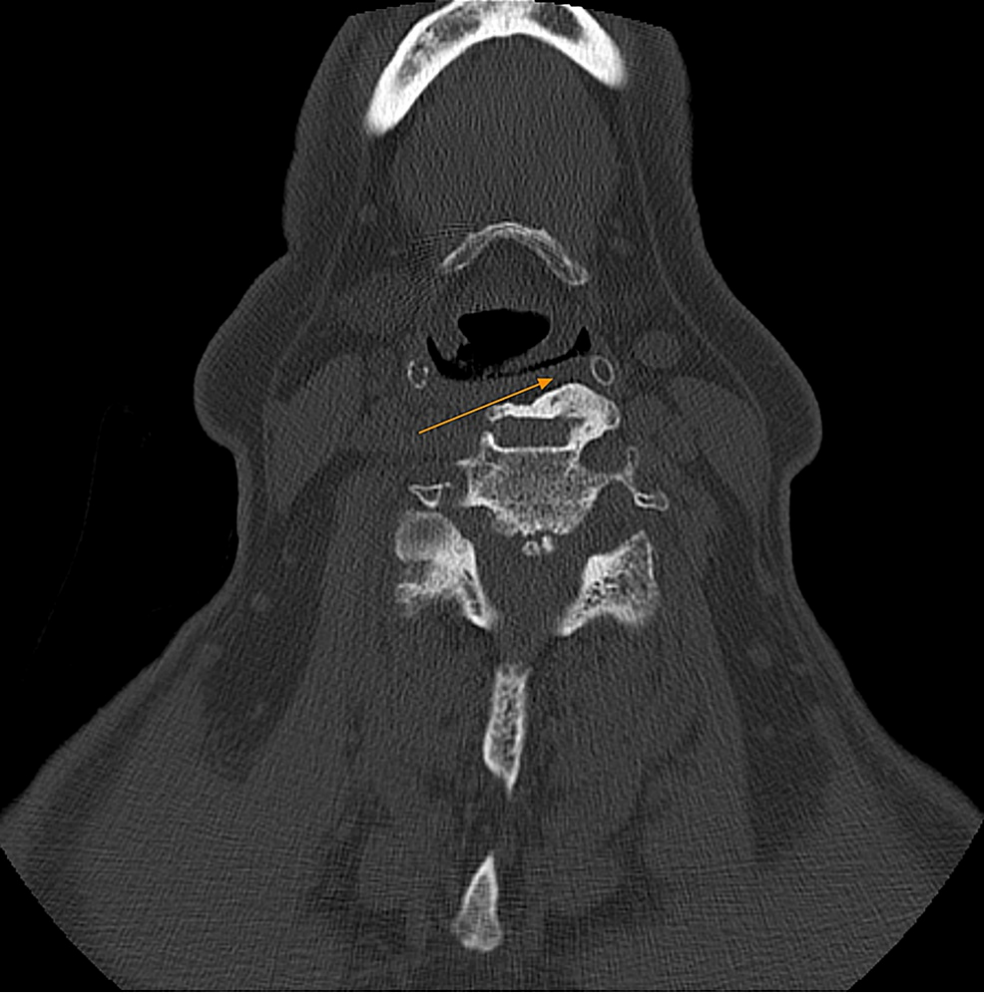
Transverse computed tomography demonstrating a bony bridge between adjacent osteophytes, causing a left impression on the airway. The arrow pointing to the osteophytes at the C2-C3 vertebral level.

Following his admission, a computed tomography (CT) scan was performed. CT scan evaluation was invaluable in revealing the presence of large mass-occupying cervical osteophytes which resulted in the vocal cord paresis (Figure 2).

**Figure 3:**
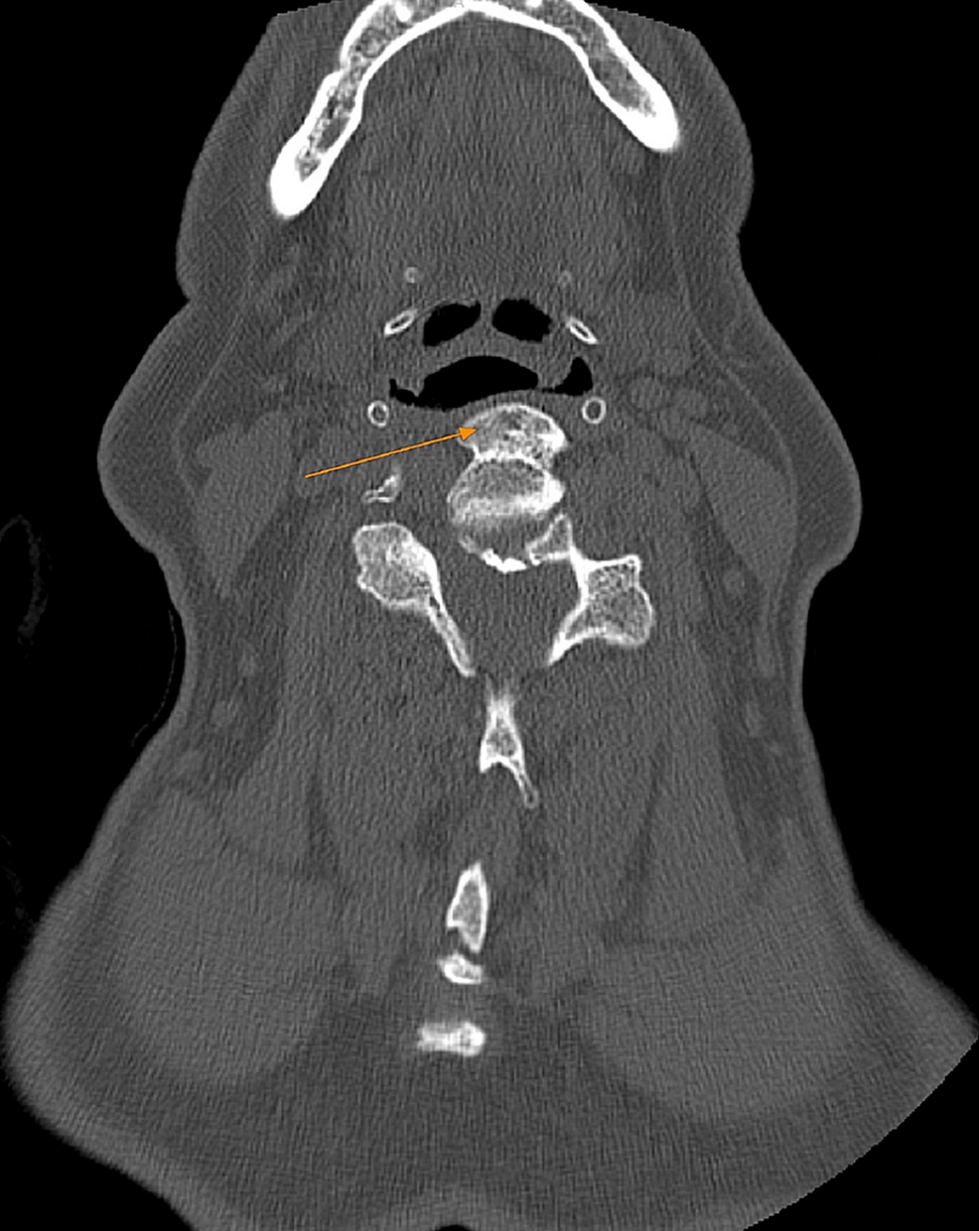
Transverse computed tomography demonstrating flattened anterior osteophytes which cause invasive phenomena and compress the upper airway. The arrow pointing to the osteophytes at the C3-C4 vertebral level.

**Figure 4:**
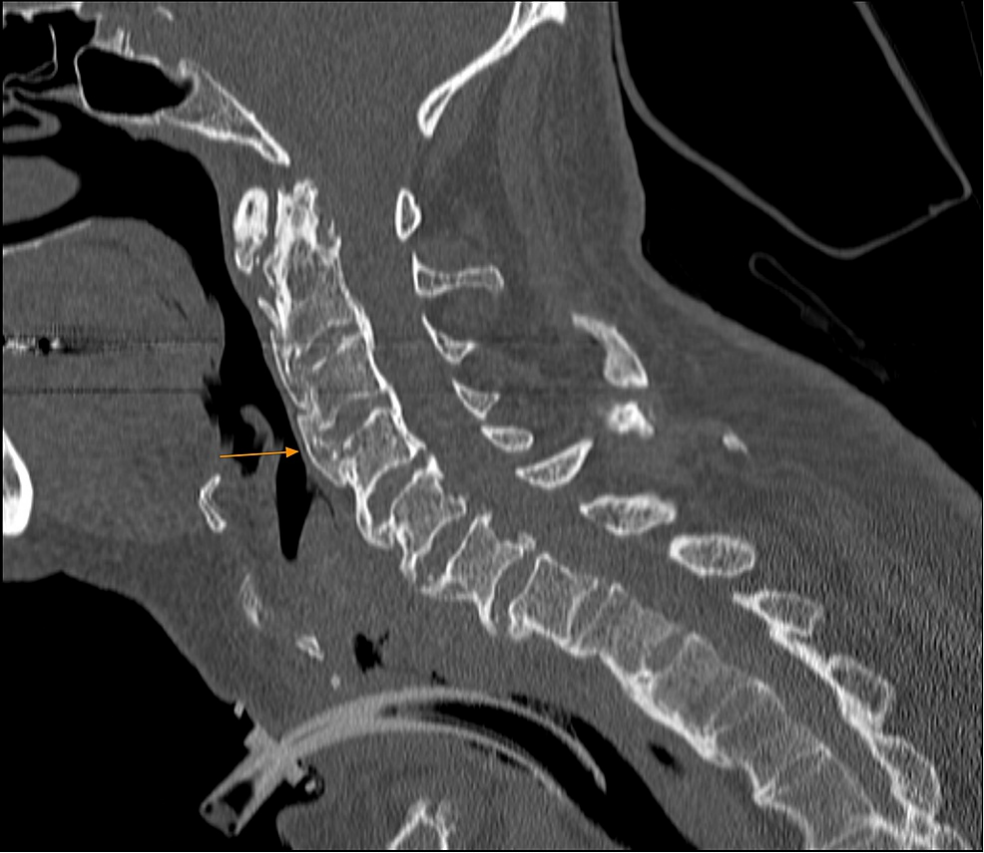
Preoperative sagittal cervical computed tomography demonstrating bridging of the vertebral bodies. The arrow pointing to the bridging of the anterior wall of the C3-C4 vertebral level.

Furthermore, two days post-tracheotomy, microlaryngoscopy was performed to collect biopsies from the left arytenoid, the osteophytes behind the larynx, and the posterior-left wall of the upper oesophagus (Table 1). The biopsies revealed low-grade epithelial dysplasia of the laryngeal mucosa, the presence of mature bone (Figures 3, 4), while inflammatory changes and a focal abscess were found on the larynx. No malignant features were present.`

**Table 1:** Location of biopsy samples and their histopathologic results.

Biopsy Location	Results
Left arytenoid	Low-grade epithelial dysplasia of the laryngeal mucosa
Osteophytes behind the larynx	Presence of mature bone (osteophytes)
Posterior-left wall of the upper oesophagus	Inflammatory changes and a focal abscess

After consulting neurosurgeons, the patient had an operation to remove part of the osteophytes that were compressing the larynx and displacing the oesophagus. Through an anterior cervical approach, excision of osteophytes from C3-C7 vertebral levels was performed. The patient’s clinical condition improved, and his vital signs were normalized. Postoperatively, his heart rate was 60 beats/minute, respiratory rate was 12 breaths/minute, and his oxygen saturation was 98%. 

However, upon an attempt at oral feeding, the patient was found coughing up food. Food was identified in the area around the tracheostomy tube. Esophagoscopy, bronchoscopy, and a barium swallow test were performed. A tracheo-oesophagal perforation was discovered between the upper part of the oesophagus and the trachea. Subsequently, a gastrostomy tube was placed for feeding. Finally, the patient recovered and the back pain was relieved. He was discharged with the tracheostomy and gastrostomy tubes until their removal was deemed medically possible. Unfortunately, eight months postoperatively removal is not feasible.

## Discussion

DISH syndrome is an uncommon non-inflammatory musculoskeletal condition, which is more prevalent in the elderly population [2]. The characteristic sequential osteophytes in the axial skeleton are the hallmark of the condition. The diagnosis of DISH syndrome is based on three findings: flowing osteophyte presence in no less than four contiguous vertebrae, few or no degenerative disk changes to exclude degenerative spondylitis and the lack of apophyseal joint ankylosis, and the absence of erosions and fusion at the sacroiliac joint to exclude ankylosing spondylitis (AS) [3]. It is thought that DISH pathogenesis is polygenic and influenced by the interaction of many gene variants and environmental factors. The DISH phenotype consists of potential disturbances in various genes with different chromosomal localizations and expressions [6].

Osteophytes in the cervical spine of our patient led to some unusual complications such as dysphagia and vocal cord paresis. However, osteophytes in the cervical vertebrae are commonly asymptomatic, which leads to a delay in diagnosis [2]. Dysphagia is a complication presenting in 0.2%-28% of patients with DISH syndrome [6]. The presence of osteophytes at C3-C7 anteriorly compress, and if large enough, displace the oesophagus, leading to dysphagia, our patient’s presenting complaint [7]. The patient’s second cervical complication, the vocal cord paresis, is a rare complication of DISH. The cause of the paresis is compression of the nerves supplying the vocal cords, such as the recurrent laryngeal nerve and laryngeal nerve, both branches of the vagus nerve [8].

An important clinical feature of the patient’s medical history was the presence of chronic back pain. The differential diagnosis for back pain ranges from mechanical and degenerative aetiologies to inflammatory and infectious causes [9]. Consequently, back pain is not always easy to diagnose but is very frequently the presenting complaint [10]. Chronic back pain with minor or no trauma on an older patient raises clinical suspicion for DISH syndrome and warrants further investigation [1]. 

The simultaneous tracheo-oesophagal perforation following anterior cervical surgery is an extremely rare complication [11]. While oesophagal perforation is a known complication of anterior cervical surgery, tracheal injury is thought to result from pressure in the posterior tracheal wall, a less common complication of anterior cervical surgery [11,12]. Tracheo-oesophagal perforations can be treated conservatively or operatively [11]. The choice of treatment depends on the location, size, and factors such as mechanical ventilation, a contraindication for surgical repair [12].

Moreover, the diagnosis of DISH syndrome is challenging. Autoantibodies and inflammatory markers were commonly seen in other musculoskeletal conditions such as rheumatoid factor, antinuclear antibodies, and C-reactive protein are negative in DISH syndrome [1]. The most definitive test is imaging [1]. However, comorbidities associated with DISH syndrome are frequently utilized to aid in the diagnosis. These include obesity, hyperlipidemia, hypertension, hyperuricemia, and diabetes mellitus [2].

Treatment of DISH syndrome is based on symptomatic therapy [13]. Analgesics, NSAIDs, muscle relaxants, diet management, and exercise therapy have all effectively managed patients with DISH syndrome [14,15]. However, exercise therapy was found effective only in the lumbosacral region [16]. An interesting management option described in the literature is the cervical collar. The use of a cervical collar was associated with improved outcomes in patients who underwent surgery for anterior cervical osteophytes [17]. Surgery is recommended when conservative treatment fails to improve quality of life and for patients with severe cervical symptoms such as dysphagia and airway obstruction [2]. Surgery is rarely utilized. The only two instances when surgery has been proven beneficial include the presence of large cervical osteophytes and lumbar spinal stenosis [13]. Nonetheless, even if removing the osteophytes relieves mechanical pressure and symptoms subside, re-emerging osteophytes, years after the surgery, have been described in the literature [18].

In advanced and insidious cases of DISH syndrome, tracheostomy is a lifesaving procedure that reverses airway obstruction. The tracheostomy is vital in stabilizing the patient for treatment to be initiated. The indications for emergency tracheotomy include acute upper airway obstruction, aspiration of a foreign body, and penetrating laryngeal trauma, among others [19].

Finally, DISH syndrome poses a diagnostic challenge as it mimics the differential diagnosis of carcinoma of the larynx. Carcinoma of the larynx has a mean age of 65 and smoking is a significant risk factor for oncogenesis. It initially presents with hoarseness and pain with swallowing, and late symptoms include dysphagia and airway compromise which are all characteristics that are found in DISH syndrome [20].

## Conclusions

DISH syndrome is a rare musculoskeletal condition. Early diagnosis and close follow-up are the gold standards for the management of DISH syndrome. The close monitoring of the osteophytes' growth is vital to prevent damage such as vocal cord paresis, changes in the anatomy of the larynx, and airway obstruction resulting in a potential emergency tracheostomy.
